# Evolutionary development of embryonic cerebrospinal fluid composition and regulation: an open research field with implications for brain development and function

**DOI:** 10.1186/s12987-016-0029-y

**Published:** 2016-03-15

**Authors:** David Bueno, Jordi Garcia-Fernàndez

**Affiliations:** Department of Genetics, Microbiology and Statistics, Unit of Biomedical, Evolutionary and Developmental Genetics, Faculty of Biological Sciences, University of Barcelona, Av. Diagonal 643, 08028 Barcelona, Catalonia Spain

**Keywords:** Neuro-evo-devo, Embryonic cerebrospinal fluid (eCSF), Blood-eCSF interface, Primary neurogenesis, Neural progenitor cells, Brain development, Cephalic vesicles

## Abstract

Within the consolidated field of evolutionary development, there is emerging research on evolutionary aspects of central nervous system development and its implications for adult brain structure and function, including behaviour. The central nervous system is one of the most intriguing systems in complex metazoans, as it controls all body and mind functions. Its failure is responsible for a number of severe and largely incurable diseases, including neurological and neurodegenerative ones. Moreover, the evolution of the nervous system is thought to be a critical step in the adaptive radiation of vertebrates. Brain formation is initiated early during development. Most embryological, genetic and evolutionary studies have focused on brain neurogenesis and regionalisation, including the formation and function of organising centres, and the comparison of homolog gene expression and function among model organisms from different taxa. The architecture of the vertebrate brain primordium also reveals the existence of connected internal cavities, the cephalic vesicles, which in fetuses and adults become the ventricular system of the brain. During embryonic and fetal development, brain cavities and ventricles are filled with a complex, protein-rich fluid called cerebrospinal fluid (CSF). However, CSF has not been widely analysed from either an embryological or evolutionary perspective. Recently, it has been demonstrated in higher vertebrates that embryonic cerebrospinal fluid has key functions in delivering diffusible signals and nutrients to the developing brain, thus contributing to the proliferation, differentiation and survival of neural progenitor cells, and to the expansion and patterning of the brain. Moreover, it has been shown that the composition and homeostasis of CSF are tightly controlled in a time-dependent manner from the closure of the anterior neuropore, just before the initiation of primary neurogenesis, up to the formation of functional choroid plexuses. In this review, we draw together existing literature about the formation, function and homeostatic regulation of embryonic cerebrospinal fluid, from the closure of the anterior neuropore to the formation of functional fetal choroid plexuses, from an evolutionary perspective. The relevance of these processes to the normal functions and diseases of adult brain will also be discussed.

## Background

From its embryonic beginnings and throughout adult life, the vertebrate brain is organised around an extraordinarily dynamic and complex fluid called cerebrospinal fluid (CSF). The study of CSF attracts growing interest in brain development research, as it is an active signalling medium containing growth factors and signalling molecules involved in the regulation of multiple cell functions in the central nervous system (CNS), including brain development, homeostasis and disease (for general reviews, see [1–7]). Today, several research findings have generated sufficient evidence to support the hypothesis that embryonic CSF (eCSF) is involved in nearly all aspects of embryonic brain development.

Evo-devo (evolutionary development) has flourished in the last 20 years, since initial studies were published in the 1980s by Nobel Laureates Edward B. Lewis, Christiane Nüsslein-Volhard and Eric Wieschaus. In these studies, some of the genes important in *Drosophila* fruit fly development, for example the *Hox* genes, were found to be broadly conserved among metazoans [8–10]. Even functions of these genes appeared to be conserved, as *Hox* vertebrate genes could substitute, at least partially, for missing homolog *Drosophilahox* genes. More recently, variation in the nervous system of metazoans has also begun to be examined from an evo-devo perspective. This has led to the emergence of the term evo-devo-neuro [11] or neuro-evo-devo [12]. A nearly ubiquitous theme in evo-devo studies on CNS is the search for cellular and/or molecular causes of evolutionary change in development trajectories. Just as the field of evo-devo has shed light on how diversity in animal body plans are variations on a few developmental themes [13, 14], so the study of neuro-evo-devo is beginning to illuminate how morphological and functional diversity in the brain are variations on developmental and neurochemical themes [15, 16].

**Fig. 1:**
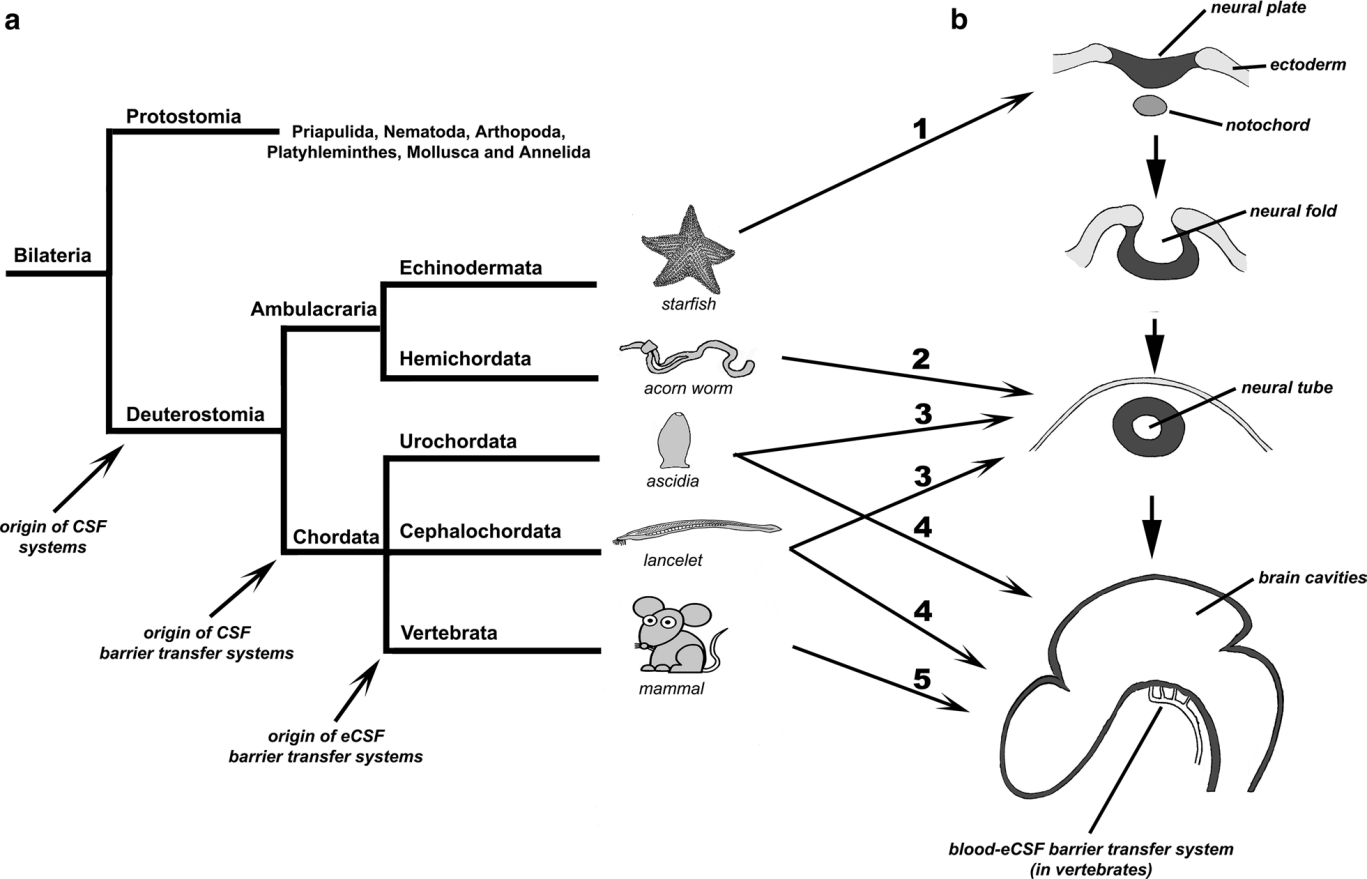
Phylogeny and ontogeny of the CNS. **a** Phylogenetic relationships among main deuterostome clades, as cited in the text. **b** Ontogeny of the CNS, including the brain, in vertebrates. The origin of the different elements of the CSF system has been indicated. Similarities between deuterostome clades and CNS development in vertebrates have also been indicated. *1* Adult starfish possess a neural plate-like nervous system similar to one from early vertebrate embryos, before the formation of the neural tube; *2* Adult Hemichordata possess a hollow dorsal nerve chord similar to one from vertebrate embryos before the closure of the anterior neuropore; *3* Urochordata and cephalochordata larvae possess a hollow dorsal nerve chord anteriorly open similar to one from vertebrate embryos before the closure of the anterior neuropore; *4* Urochordata and cephalochordata adults possess a closed dorsal nerve chord similar to one from vertebrate embryos after the closure of the anterior neuropore; *5* In vertebrates, the neural tube becomes a physiologically sealed system from a very early developmental stage and the site of the embryonic transfer system is depicted

Most of molecular evo-devo research has focused on the functional comparison of homologous genes. In this respect, the genes that specify brain patterning early in development are highly conserved in bilaterians [17–20]. A comparative analysis of these genes in different taxa has provided insights into the early origin of the central nervous system and regional brain homologies in vertebrates and invertebrates. For example, all known bilaterian nervous systems are established via transforming growth factor β (TGF-β) family signalling, which arranges dorsoventral polarity, except in nematodes [17, 21, 22]. However, the chordate nervous system is dorsoventrally inverted compared with protostomes, and it has been suggested that hemichordates represent an intermediate body plan [23] (see Fig. 1 for a general cladistics phylogenetic tree). Similarly, anterior–posterior patterning is in part defined by *Otd/Otx* (anterior) and *Unpg/Gbx* (posterior) expression, with *Pax2/5/8* expressed at the intersection [24–26]. Likewise, comparisons of patterning genes after the establishment of the anterior–posterior axis have identified evolutionary relationships among brain regions in bilaterians from very distant taxa. For example, expression of *Pax6* and *Emx1* specify the pallial telencephalon, whereas *Dlx* and *Nkx2.1* specify subpallial regions [27, 28] (for a review, see [12]). Interestingly, invertebrate mushroom bodies have similar evolutionary origins to the pallium of vertebrates, as annelid mushroom bodies are also specified by *Pax6* and *Emx1* expression [29].

We will not continue with this line of argument as it is beyond the scope of this paper, except to cite another example of classic evo-devo research related to brain evolutionary development that has been analysed by the authors of this review and their collaborators to which we will return later. A comparison of the structure and expression of *Evx* genes in the cephalochordate amphioxus (which belongs to the sister group of vertebrates) and its vertebrate homologs linked invertebrate and vertebrate *Evx* functions across different taxa, and pointed to crucial evolutionary vertebrate innovations for brain development at the level of molecular and morphological regionalisation [30]. Amphioxus has been reported to have two *Evx* genes that are genomically linked. *AmphiEvxA* is prototypical to the vertebrate *Evx1* and *Evx2* genes with respect to its sequence and expression, whilst *AmphiEvxB* is very divergent. Mapping the expression of *AmphiEvxA* onto a phylogeny showed a role in gastrulation, dorsal–ventral patterning, tailbud formation and neurogenesis. This suggests that basic *Evx* function has been retained throughout bilaterian evolution. Interestingly for brain evolutionary development, *AmphiEvxA* is not expressed in amphioxus in the region homologous to the vertebrate midbrain–hindbrain boundary, a well-known organising centre for brain development that is also called the isthmic organiser (IsO). This observation is consistent with the elaboration of the full organiser properties of this region being a vertebrate innovation. Later in this paper, we will go back to this organising centre, as its maintenance during early embryonic brain development also depends on the actions exerted by the eCSF [31].

To cite a final example, in this case also related to some aspects of animal behaviour, it has been reported that gonadotropin-releasing hormone, which is produced by the pituitary gland located at the base of the brain in vertebrates, is also produced in the most anterior part of the amphioxus body. This hormone is a key player in the control of vertebrate reproduction, and it has been shown to display seasonal variations in the Cephalochordate amphioxus with respect to their reproductive cycle. This suggests that it plays an ancient role in the control of reproduction in all chordates, even before the evolution of a defined pituitary gland [32]. However, despite the explosion of evo-devo studies on some morphological traits, the impact of behavioural factors upon speciation has scarcely been analysed [11], although it has been said that the elaborate and complex nervous system of amniotes is correlated with their behavioural repertoire [33].

Similarly, very little work has been done on brain cephalic vesicles from the evo-devo perspective, and the same is true for both CSF in general and eCSF in particular. In this regard, from fetal stages through adult life, it has long been known that CSF is primarily produced by choroid plexuses. It has been shown that choroid plexus is derived from the same embryonic primordium as the hindbrain roof plate, and that it develops in a patterned, segmental manner, from lineage-restricted compartments expressing Wnt1 [34]. Moreover, it has also been shown that molecular heterogeneity between telencephalic and hindbrain choroid plexi contributes to region-specific, age-dependent protein secretion in vitro, which may contribute to dynamic signalling gradients across the mammalian cerebroventricular system [35]. However, eCSF has a role in vertebrate brain development at a much earlier stage, at least just after the closure of the anterior neuropore and before the initiation of brain primary neurogenesis, prior to the existence of a functional choroid plexus (for general reviews, see [1, 2, 4]).

The main objective of this review is to demonstrate that the evolutionary development of the embryonic brain cavity system, including the eCSF it contains and the mechanisms controlling its formation and homeostasis, must also be taken into account to get a better overall understanding of brain development, CNS evolution, and neurological diseases. To present our thesis in an ordered way, we will first review data on evolutionary changes of the brain cavities in metazoans, primarily in the deuterostome lineage. We will then review research on eCSF function and composition, and on its formation and homeostasis regulation, much of which has been developed by the authors and their collaborators over the last 10 years. Finally, we will integrate all these data in a comprehensive thesis about the actual importance of the developmental evolution of the brain cavities and eCSF, in the light of mammalian brain development and human diseases, with some comments on behavioural aspects.

### Embryonic CSF evolutionary aspects: from the origin of deuterostomes to vertebrates

In vertebrates, brain formation begins early during development. Generally speaking, its development involves several distinct phases (Fig. 1; for a general description of central CNS development, see [36–38]). First, a portion of the dorsal ectoderm develops into neural ectoderm and forms the neural plate, which is in contact with the amniotic fluid in amniotes (reptiles, birds and mammals) or with the surrounding water in the anamniotes (fish and amphibians). Second, the neural plate folds longitudinally to form the neural tube, which becomes the rudiment of the CNS, trapping some of the surrounding fluid within its lumen. The tube still remains in contact with the environmental fluids through the open neuropores. Then, the neural tube polarises into a posterior spinal cord and an anterior expanded brain. Initially, the embryonic brain is a hollow fluid-filled vesicle surrounded by a pseudo-monostratified neuroepithelium. The subsequent closure of the anterior neuropore enables the organism to exert some control over CSF formation and composition for the first time. Thus, the CSF composition can become independent of environmental fluids. Finally, histogenesis of the neuroepithelium occurs. Interestingly, from the evo-devo perspective, this developmental process parallels evolutionary aspects of brain cavities and CSF formation and regulation in the deuterostome metazoan lineage (Fig. 1).

Metazoan phylogeny classifies the vertebrata as a subphylum of the phylum Chordata, which also includes two other subphyla, the Urochordata and the Cephalochordata. Within this scheme, the phylum Chordata is included in a major group, the Deuterostomia, which also includes the phyla Echinodermata and Hemichordata. These last two phyla are in turn grouped in the clade Ambulacraria. Chordates invariably possess a notochord and a dorsal neural tube, and share tadpole-type larvae containing the notochord and a hollow nerve cord. However, Ambulacrarians have dipleurula-type larvae containing a hydrocoel. It has been proposed that the evolutionary occurrence of tadpole-type larvae with a hollow nerve chord was fundamental to the origin of chordates [39]. The sister group of Deuterostomia are the Protostomia, including the clades Priapulida, Nematoda, Arthopoda, Platyhelminthes, Mollusca and Annelida, none of which exhibit a hollow nerve tube with an internal lumen or brain cavities.

It is thought that the CSF system evolved in the deuterostome lineage from the ancestor of Ambulacraria and Chordata, as a way to maintain the chemical environment required for the functioning of CNS cells, including the neuroendocrine pathways [40]. In this context, it has been said that the system of CSF-contacting neurons has a special role in taking up, transforming and emitting nonsynaptic signals mediated by this brain fluid. Most CSF-contacting neurons send dendritic processes into the CSF of the brain ventricles or central canal, where they form terminals bearing stereocilia. The axons of these CSF-contacting neurons transmit information taken up by dendrites and perikarya to synaptic zones of various brain areas. The importance of nonsynaptic signal transmission in the function of nervous tissue has been confirmed, and it has been considered that its evolutionary origin could be the deuterostome ancestors, before the split between Ambulacraria and Chordata clades (reviewed by [41]).

Echinoderms, such as starfish, have a neural plate-like nervous system. The neurons of the radial nerves and “brain” are always in contact with seawater, which allows them to directly detect its chemical composition and other physical parameters such as water temperature, and modulate the activity of the nervous system and overall animal behaviour accordingly (Fig. 1) [40, 42–44]. However, the open nature of this neural plate-like nervous system means that it cannot respond to neuroendocrine or other molecular signalling pathways. In contrast, Hemichordata, such as the acorn worm *Saccoglossus kowalevskii*, which may represent an intermediate body plan [23], exhibit both dorsal and ventral nerve cords. While the ventral cord runs only as far as the collar, the dorsal cord reaches into the proboscis, and is often hollow.

Regarding the phylum Chordata, the development of the cephalochordate amphioxus *Branchiostoma lanceolatum,* includes a larval stage in which the neural plate is folded longitudinally to form a neural tube, but the anterior neuropore remains open throughout this stage. This allows seawater to enter the ventricular lumen of the lancelet larval “brain”. Thus seawater represents the first “internal” fluid environment of lancelet brain which consequently depends directly—but not necessarily exclusively—on the external environment [40, 41]. In these organisms, nearly all neurons are found to be in direct contact with the internal fluid of the neural tube-like central nervous system. However, when lancelets reach adulthood, the anterior neuropore closes and the ventricular lumen of the “brain” becomes a closed system (Fig. 1). Thus, the composition of this fluid no longer depends directly on the surrounding water environment, but largely on the activity and metabolism of the organism. This modified sea water constitutes the primary CSF. At this stage, the regulatory function of CSF-contacting neurons likely become important through their interaction with the metabolic state of the brain tissue [44–46].

Similarly, the development of the Urochordate ascidia *Ciona intestinalis,* includes a larval stage in which the neural plate is folded longitudinally to form a neural tube, but the anterior neuropore also remains open (Fig. 1). Unlike lancelet larval development, ascidians follow an invariant pattern of embryonic cleavage, and their larval CNS possess fewer than 400 cells, most of which are in direct contact with the internal fluid of the neural tube-like central nervous system, as revealed by cell lineage studies [47–49]. Nevertheless, as in Cephalochordates, when ascidians reach adulthood the anterior neuropore closes and the ventricular lumen of the “brain” becomes a closed system, which includes a sensory vesicle (Fig. 1).

In lancelets and ascidians, nearly all neurons contact the lumen of the central nervous system. Consequently, it has been suggested that the CSF-contacting neurons of vertebrates are derived from an ancient epithelial neuron-type of the ectoderm, and thereby represent a phylogenetically old cell type, the “protoneuron” [50]. In mammals, only the CSF-contacting neurons of the spinal cord retain their ancient structure; the rest of the neurons no longer contact CSF [41].

Finally, in vertebrates, the anterior neuropore closes very early in neural tube development, just before primary neurogenesis starts, and thus the composition of the CSF contained within the cephalic cavities depends exclusively on the surrounding tissue and on the rest of the embryo from this stage. Environmental conditions are no longer directly detected by CNS neurons, and this function is passed to specific sensory organs. This opens up the possibility of precisely regulating CSF composition and homeostasis. The gain in CSF regulation implies, or has been paralleled by, a gain in morphological and physiological neural complexity. Thus, it has been reported that, at least in mammals, CSF is essential for the formation of the layers of neurons [51, 52] in the evolutionary progressively enlarged cerebral cortex, including the highly developed neocortex [53], a brain region that is absent in fish and amphibians (anamniotes).

CSF composition and homeostasis in vertebrate fetal and adult brains are tightly regulated by the choroid plexus, whose epithelial cells establish a blood-CSF barrier [54, 55] (reviewed by [56]). The choroid plexus is a vascular structure in the brain ventricles that secretes CSF by promoting the transport of certain molecules and electrolytes from blood plasma and producing others that are delivered directly to the ventricles [57–61]. All vertebrates analysed, from fish to mammals, possess a set of choroid plexuses [40, 56] and it has been described that the choroid plexus increases in complexity among the various vertebrate classes. However, no choroid plexus has been detected in non-vertebrate chordates, such as Cephalochordates. Acting in parallel, CNS homeostasis in adult vertebrates is also controlled by the blood–brain barrier (BBB) vessels in the brain, which significantly impede entry from the blood to the brain of virtually all molecules, except those that are small and lipophilic. However, BBB vessels also allow sets of small and large hydrophilic molecules, e.g., gene products, to enter the brain via various transport processes (reviewed by [62]). Control of eCSF production and homeostasis in vertebrate embryos from the closure of the anterior neuropore to the formation of functional choroid plexuses during fetal stages has not been analysed until recently. This developmental stage is particularly interesting for brain formation, since it is mainly characterised by rapid brain anlagen growth and initiation of primary neurogenesis in the neural progenitor cells lining the cavities.

### Embryonic CSF function: from neural progenitor cell functions to the establishment of organising centres

CSF has intrigued philosophers, physicians and scientists for a long time. The earliest mention of a fluid within the brain dates back to ancient Egypt [5], and the first studies of barriers in the brain are attributed to Herophilus (335-280 BCE), who described the choroid plexus. The first reports on CSF functions are attributed to Galen of Pergamon (129-200/216). Studies on eCSF are much more recent, dating back to the mid-twentieth century. Due to its specific molecules and mechanical properties, it has been determined that eCSF plays several crucial roles during the early stages of brain development and may influence adult brain function.

Using several experimental approaches, mainly in zebrafish, chick and mouse embryos, it has been reported that the progressive increase in eCSF volume exerts positive pressure against the neuroepithelial walls and generates an expansive force, which contributes to brain expansion [63–65]. Moreover, as proteoglycans are the major components of the extracellular matrix found in embryo brain cavities, it has been suggested that the special osmotic properties of chondroitin sulfate proteoglycan and other proteoglycans in eCSF may cause water retention in the cavities [66, 67]. This would contribute to the generation and regulation of inner cephalic hydrostatic pressure.

Studies on in vitro cultures of neuroectodermal explants from chick and rat embryos at E4 and E12.5 (E stands for embryonic development day) respectively, which correspond to the stage at which the anterior neuropore has just closed and brain primary neurogenesis is initiated, have shown that diffusible molecules contained within eCSF, including growth factors and cytokines, contribute to the regulation of some basic neural progenitor cell functions, i.e., their survival, proliferation, and differentiation [68–70]. Furthermore, it has been reported that neuroectodermal tissue explants cultured with only a chemically defined medium show lower numbers of proliferating cells, an increased number of apoptotic cells, and a severe decrease in the number of cells engaged in the process of neural differentiation compared to control embryos. However, when the medium is supplemented with eCSF, these basic cellular parameters remain close to those of embryos developed in ovo (for chicks) or in utero (for rats). It has also been reported that repeatedly draining eCSF from embryonic zebrafish brain vesicles not only prevents brain enlargement due to the lack of hydrostatic pressure (see paragraph above), but also decreases the rate of cell survival [63], paralleling the effects reported in amniotes (at least in birds and mammals). Moreover, the particular effect of the eCSF on neural progenitor cells depends on the developmental stages from which both the fluid and the cells have been obtained. This suggests that the composition of the eCSF varies with time, as does the receptiveness of the cells, most probably through developmentally regulated specific receptors [52, 71, 72].

Most of the proteins identified in the eCSF (see the section below on *eCSF protein content*) have known physiological functions during embryonic development in systems other than CSF that nevertheless are consistent with the overall roles reported for eCSF during CNS development [73, 74]. Thus, functional in vivo and in vitro analysis of some of these molecules at the beginning of primary neurogenesis, before the formation of functional choroid plexuses, has revealed specific roles in the function of eCSF in neuroepithelial progenitor cell behaviour. For example, it has been reported that immunoblocking of active FGF2 in eCSF in chick embryos severely disrupts neuroepithelial stem cell proliferation and differentiation [75]. Similarly, it has been reported that the LDL lipid fraction, transported by apolipoprotein B in eCSF, is also involved in regulating neuroepithelial progenitor cell proliferation and differentiation in the same system [76].

Other reports in chick embryos have shown that retinol-binding protein (RBP) is responsible for transporting all-trans-retinol from the embryonic plasma to the eCSF (as detected by high-performance liquid chromatography), from where it reaches the neuroepithelium to be transformed into retinoic acid by the retinoic acid-synthesising enzymes expressed in this embryonic tissue; and that the presence of these molecules is necessary both in vivo and in vitro to maintain basic neuroepithelial cell parameters (i.e., proliferation and differentiation [77, 78]). Interestingly, a parallel situation has been described recently in zebrafish embryos [79]. This suggests that at least some of the basic mechanisms in which eCSF is involved during early brain development are conserved among vertebrates. It has also been shown that large glycoprotein SCO-spondin contributes to the control of neuroepithelial cell proliferation via eCSF in chick embryos [80], and that eCSF contained in nanovesicles carrying evolutionarily-conserved molecules promotes neural stem cell proliferation [81]. Other findings include the capacity of eCSF to activate neurogenesis of neural precursors within the subventricular zone of the adult mouse brain [82].

Therefore, eCSF contains specific molecules that affect neural progenitor cell behaviour. In addition, eCSF has been reported to be involved in regulating the expression of genes that are known to be implicated in brain patterning [31]. Thus, when dorsal mesencephalic neuroepithelial explants lacking IsO (a known organising centre for mesencephalon and brain development) or explants including IsO are cultured in a chemically defined medium but in the absence of eCSF, the typical expression of dorsal mesencephalic and IsO genes, i.e., *Otx2* and *Fgf8* respectively, is disrupted. Conversely, when dorsal mesencephalic explants including IsO are cultured in an eCSF-supplemented medium, they do express these genes. Interestingly, when dorsal mesencephalic explants lacking IsO are cultured with an eCSF-supplemented medium, they also show ectopic expression domains of *Shh* in the mesencephalic neuroectoderm, even though this gene is typically expressed in the ventral, but not the dorsal, neuroectoderm. Only the concurrence of eCSF and IsO allow this tissue to mimic its typical pattern of gene expression, which suggests that IsO and some molecules in eCSF act synergistically in brain development, as is the case for the retinoic acid system [77, 78]. It is worth noting that the elaboration of the full organiser properties of this region [30] and embryonic control over eCSF composition are both vertebrate innovations that act synergistically in brain development [31].

Moreover, the presence of folate and some of its derivatives within fetal CSF has been reported [83], as well as the presence of reduced-folate carriers within the neuroepithelium from E9.0 in mouse embryos [84, 85]. Folate is known to prevent neural tube defects when supplemented prior to conception, and it has also been reported to contribute to the establishment of epigenetic modifications [86–88], which in turn are crucial for CNS development [89], including adult behaviour [90–93]. Taken together, the results of all the studies mentioned in this section point to the crucial role of this embryonic fluid in the regulation of basic neural progenitor cell function and CNS development, before the formation of a functional choroid plexus. This may have implications for adult brain function.

### Embryonic CSF protein content: from extracellular matrix components to growth factors

Classic studies performed on several species (chick embryos at developmental stages E2.5 to E6.5; sheep fetuses at E35 and E60; and rats from E12 to E22 and neonatal) showed that eCSF has a higher concentration of total proteins than adult CSF [94–100]; reviewed by [101]. Initially, individual proteins were identified based on crossed immunoelectrophoresis or by SDS-PAGE protein separation and molecular mass inference. More recent and accurate proteomic analyses of avian and mammalian eCSF have confirmed and greatly expanded most of the initial findings. The first reports to use conventional proteomic techniques (2D-electrophoresis, in-gel digestion, and ESI–MS/MS mass spectrometry analysis) were carried out on chick and rat eCSF at E4 and E12.5, respectively, which corresponds to the developmental stages when there is maximum neuroepithelial progenitor cell proliferation, and coincides with the beginning of brain neural differentiation. These studies showed the presence of dozens of specific proteins within this fluid, most of which may be involved in brain development, by inference from their roles in systems other than CSF [73, 74]. They include gene products involved in extracellular matrix, osmotic pressure and ion carriers, cell death and quiescence, apolipoproteins, retinol and vitamin D carriers, and antioxidant and antimicrobial products, among others (for a comparative review, see [101]).

From the evolutionary point of view, the most remarkable dissimilarity between chick and rat eCSF proteomes is the presence of enzymes and enzyme regulators in rat eCSF, as well as an increased number of members of the apolipoprotein family. Only two different apolipoproteins have been identified in chicks, AI and AIV, whereas at least five have been found in rats, including AI and AIV, as well as B, E, and M. These differences may be related to the higher complexity of the CNS in mammals, although apolipoprotein B was later identified in chick eCSF by Western-blot analysis using specific antibodies [76]. These dissimilarities may also be due to differences in the timing of choroid plexus formation between the two species. In this regard, E4.5 chick embryos correspond to a developmental stage that is 2.5 days before the first appearance of the choroid plexus. However, in E12.5 rat embryos, transthyretin-positive choroid plexus primordium starts to be detected, although it is probably not fully functional, as suggested by the coexistence of a specific, temporary embryonic blood-eCSF barrier [102] (see the section below on *eCSF composition and regulation*).

An extensive and thorough proteome analysis of rat and human eCSF from several different stages and locations of brain vesicles [i.e., E12.5, E14.5 from both the lateral and fourth ventricles, and E17.5 during cortical development in rats; and Carnegie Stage 20 (CG20) in humans] has also been reported [103]. They identified 423, 318, 249, and 382 proteins, respectively, in rats, and 188 proteins in humans, 130 of which are shared with rats. Although differences in protein number between rat and human eCSF has not been fully explained, it has been suggested it may be due to the difficulty in experimentally matching stages of embryonic cortical development between humans and rats [103], as the composition of this fluid has been proved to be highly dynamic. The categorisation of these proteins based on their molecular function and action on biological processes are almost identical, which suggest that they represent essential eCSF functions. They include several growth factors and cytokines. Similar results were obtained more recently by Bueno, Miyan and Parvas on eCSF from chick and rat embryos at E4 and E13 respectively, using a high-performance liquid chromatography ion-trap column coupled to an ESI MS/MS mass spectrometer, which is a more sensitive technique (unpublished results).

Other studies have examined the presence of growth factors and cytokines within eCSF by immunohistochemical procedures, since these molecules, crucial for controlling developmental processes, exert their actions at very low concentrations, and thus are not usually detected by conventional proteomic techniques. For example, Western-blot analyses of both avian and mammalian eCSF using specific antibodies have revealed the presence of fibroblast growth factor 2 (*Fgf2*) [75], epidermal growth factor (*Egf*) [104], and leukemia inhibitory factor [105], which are known to be involved in the regulation of a number of developmental processes. Taken together, these studies, whose overall results are relatively similar, have proved to be complementary. They reinforce the putative capacity of eCSF to influence the behaviour of neuroepithelial cells and CNS development through its constituent molecules, including behavioural aspects of adult brain function.

It has been reported that a number of neurological and neurodegenerative diseases, including dementia and schizophrenia, involve alterations in CSF protein content, which in some cases is also being used in diagnostics (see, for example, [101, 106–110]). It is tempting to speculate that abnormal eCSF composition early during CNS development may favour the appearance of these diseases during adulthood, due to interference with developmental mechanisms.

### Regulation of embryonic CSF composition: the importance of the blood-eCSF barrier for controlling eCSF homeostasis in early brain development

As discussed in previous sections, the evolutionary development of brain cavities in vertebrates has provided the opportunity to tightly control eCSF composition and homeostasis from very early brain development stages. This in turn may allow this fluid to act as a mechanism for establishing neuroendocrine pathways and enabling a progressive increase in brain complexity. As described above, this hypothesis is supported by the particular effect of the specific molecules on neuroepithelial progenitor cell behaviour and on the establishment of organising centres, which are crucial for CNS development and functionality. The hypothesis is also supported by the molecular content of eCSF, as revealed by proteomic analyses and other technical approaches. However, to achieve this, barrier systems are required to control eCSF formation, composition and homeostasis from early brain developmental stages.

In fetuses and adults, there are several barrier systems that control the internal environment of the brain, such as the blood–brain barrier between the lumen of cerebral blood vessels and the brain parenchyma, the blood-CSF barrier between choroid plexus blood vessels and adult and fetal CSF, and the outer CSF-brain barrier between the subarachnoid space and overlying structures. The integrity of these barriers is crucial, as both the stability and specificity of this fluid environment are essential for normal brain development and function [111]. The barriers significantly impede the passage of virtually all molecules from the blood to the CSF, except for small, lipophilic ones, and some sets of both small and large hydrophilic molecules, such as proteins, that can enter the CSF by specific transport processes.

In avian and mammalian embryos, an inner CSF-brain barrier between the eCSF and the brain parenchyma has been reported that impedes the passage of virtually all molecules [102, 112]. However, the fact that eCSF content has proven crucial for these stages of CNS development implies the need for a physiological blood-eCSF transfer system that controls the initial composition and homeostasis of eCSF, before the formation of functional fetal choroid plexuses. This need is reinforced by the fact that most of the abundant protein fractions contained within the eCSF are produced outside of the brain anlagen, and thus they have to be transferred from the embryonic serum to the eCSF by crossing an inner eCSF-brain barrier [112]. In this context, the existence of an inner eCSF-brain barrier has been demonstrated by the microinjection of a small-sized 3 kDa tracer, biotin dextran amine(BDA3000), into the cephalic cavity, or conversely into the outflow of the heart of embryos at different embryonic days during chick development [102]. It has been shown that this inert tracer cannot cross the inner and temporary eCSF-brain barrier transfer area from day E4 on, i.e., after the closure of the anterior neuropore coinciding with the initiation of brain primary neurogenesis. However there exists a small subset of endothelial cells and adjacent neuroepithelial cells that remove the tracer from the brain cavities into the circulation. This subset of cells, which is located in the ventral mesencephalon and in the most anterior part of the ventral prosencephalon, lateral to the floor plate, may act as a temporary functional barrier transfer system, to control eCSF composition and homeostasis [102]. A number of studies published during the last 6 years, conducted primarily on chick embryos from E3 to E5 and on rat embryos at an equivalent developmental stage from E12.7 to E13.7, have demonstrated the existence of this functional, dynamic barrier transfer system. It is formed by endothelial cells of specific blood vessels and by columnar cells of the differentiating neuroepithelium. The system is active from closure of the anterior neuropore, at the beginning of brain primary neurogenesis and before the choroid plexus becomes functionally active. It contributes to the brain’s internal milieu at this crucial stage of development (see [1, 2] for a review; see Table 1 for a chronology including main aspects of embryonic blood–brain barrier transfer system in chick and rat).

**Table 1:** Chronology of the embryonic blood–brain barrier transport system formation

Morphological or physiological aspect	Embryonic day (E)	
	Chick	Rat
Closure of anterior neuropore/initial differentiation of eCSF protein content	E1.7−E2	E10.5−E11
Brain cavity becomes physiologically sealed	E3.5−E4	E12.4−E12.7
Transient embryonic blood–brain barrier starts to be physiologically functional	A bit before E4	A bit before E12.7

It has been reported that from chick E3, eCSF has a complex protein composition that differs from that of blood plasma, and that the relative concentration of these proteins varies during development and with respect to adult CSF [95–97, 99, 100]. Initially, eCSF is derived from trapped amniotic fluid. However, after the closure of the anterior neuropore, the brain cavities become physiologically sealed enabling the tight regulation of eCSF composition. Furthermore, it has been demonstrated in mice that amniotic fluid and eCSF proteomes begin to diverge during development after the closure of the anterior neuropore, and that these changes in protein composition allow neural progenitor cell self-renewal [113]. This suggests that the composition of eCSF is somehow regulated from this initial stage of brain development to fulfil specific functions on neuroepithelial progenitor cells. Various experiments have demonstrated that the transport of proteins from the blood plasma to the eCSF and vice versa in chick embryos at E4 and E5 is tightly regulated, as is the homeostatic control, which is crucial for eCSF function in brain development [102, 114]. These experiments included the quantification of endogenous chick proteins present in both the blood plasma and the eCSF (ovalbumin, retinol-binding protein, fibroblast growth factor No. 2, and immunoglobulin IgY), as well as the microinjection of several proteins of different molecular size into the brain cavities or alternatively into the outflow of the heart of chick embryos at E4 to examine the properties of this blood-eCSF barrier transfer system. The proteins used in the microinjection experiments included both endogenous chick proteins that are normally present in the eCSF or conversely not normally detected within this fluid, and proteins from sources other than chicks (bovine serum albumin, myosin heavy chain from rabbit, fibroblast growth factor No. 2, plasma retinol-binding protein, a recombinant protein from glutathione-S transferase from *Schistosoma japonicum*, alcohol dehydrogenase from *Drosophila lebanonensis*, and ovalbumin). These experiments demonstrated that the physiological ratio of chick endogenous proteins between the eCSF and the blood plasma is regulated developmentally, as the transfer rates varied from E3 to E5 according to their activity in brain development. It was also shown that the transfer of both chick endogenous proteins and microinjected proteins across the blood-eCSF interface was highly protein-specific. In other words, proteins that are not normally present within eCSF are never transferred from the blood serum to the brain cavity when microinjected into the outflow of the heart, and they are also rapidly removed from the eCSF when microinjected into the brain cavities. Interestingly, the ratio of transfer for these proteins was tightly regulated and never exceeded the physiological ratios for any developmental stage, even when the proteins were injected at levels 10- to 100-fold higher than normal physiological conditions [102].

A histological analysis of protein transfer across the blood-eCSF barrier transfer system demonstrated that it occurs only in a specific embryonic region, located in the brain stem lateral to the floor plate, in the ventral mesencephalon and the most anterior part of the ventral prosencephalon [102] (Fig. 1). This location does not coincide with the area where the fetal choroid plexus will start to develop, i.e., from an invagination of the dorsal roof plate along the midline of the neural tube. Therefore, this transient embryonic blood-eCSF barrier function appears to be independent of the choroid plexus, and probably fulfils a function that is temporarily lacking after the formation of a physiologically-sealed brain. Immunohistological analysis has also revealed that protein transport across this transient blood-eCSF barrier uses transcellular routes by means of caveolae [115]. It has also revealed the existence, in the same neuroepithelial and endothelial cells, of water and ion channels (i.e., *Aqp1*, *Aqp4*, and *Kir4.1* [116]) and glucose transporter (*Glut 1*) in both chick and rat embryos at an equivalent developmental stage [115].

### Concluding remarks

Many of the world’s first human civilisations developed along riverbanks and seashores, as they used the nearby fluid medium as a food resource. As civilisations developed over time and gained social and technological complexity, they also used the fluid pathways provided by rivers, lakes, seas and oceans to promote transport and cohesion between near and far territories. These activities included the trading of goods and news by postal services, to which people responded by adapting both their individual and collective behaviour to the changing environment. Over time, civilisations modified these fluid pathways to better serve their needs, for example by constructing harbours, dams and canals to regulate water influx, and docking for transport and irrigation. These cultural and technological human modifications of the fluid environment in some ways parallel and serve as an analogy of the evolutionary developmental changes that occur in CNS with regard to the existence of internal brain cavities filled with CSF, and with respect to CSF and eCSF composition and homeostasis regulation, as reviewed in this paper.

Thus, as human civilisations gain social complexity through the progressive use and control of fluid mediums, so does the CNS of deuterostomes with respect to the variety and complexity of neural cells and brain regionalisation. The vertebrate brain is organised, from its embryonic origins and throughout adult life, around this extraordinarily dynamic and complex fluid. CSF composition and homeostasis is tightly regulated from the closure of the anterior neuropore, just before brain primary neurogenesis starts. In contrast, in other deuterostomes, such as cephalochordates and Urochordates, CSF regulation starts during adulthood or is never present (as in Ambulacrarians). Hence, the evolutionary development of brain cavity systems, CSF, and CSF composition and regulation are milestones in vertebrate (and human) brain evolutionary development. They need to be further analysed to fully understand brain function, and may have implications for the increase in behavioural repertoire.

It will be interesting to investigate, in the light of evolution, whether the blood-eCSF embryonic barrier transfer system initially developed when brain cavities become sealed in early brain developmental stages, and thus is specific to the vertebrate lineage. Alternatively, it may represent the initial chordate evolutionary condition and may also be present in adult cephalochordates and urochordates, which have a closed cephalic cavity, but no choroid plexuses. If the latter is the case, the blood-eCSF embryonic barrier transfer system has been heterochronically reused in vertebrate embryos before the newly developed and far more complex choroid plexus is fully active.

## Conclusions

Controlling CSF composition and homeostasis is a milestone in the development and evolution of the CNS in deuterostome metazoans, especially in chordates and vertebrates. In higher vertebrates, eCSF formation is tightly regulated by a transient blood–brain barrier transfer system located ventrally in the cephalic cavities from very early stages of brain development, just after the closure of the anterior neuropore, before choroid plexus becomes physiologically functional. It is known that gene products and other molecules contained within this fluid exert crucial functions on neuroepithelial progenitor cell survival, proliferation, differentiation and patterning. The evolutionary development of these barriers and transfer systems has contributed to the increase in brain complexity. Thus, further and deeper examination of the mechanisms regulating CSF composition and homeostasis as well as the functions it exerts on neuroepithelial progenitor cells in model organisms from different deuterostome taxa may provide new and important data for the evolutionary and developmental comprehension of the vertebrate brain.

## Abbreviations

CSF: cerebrospinal fluid
CNS: central nervous system
E: embryonic day after conception
eCSF: embryonic cerebrospinal fluid
